# VEGFR2 Translocates to the Nucleus to Regulate Its Own Transcription

**DOI:** 10.1371/journal.pone.0025668

**Published:** 2011-09-28

**Authors:** Inês Domingues, José Rino, Jeroen A. A. Demmers, Primal de Lanerolle, Susana Constantino Rosa Santos

**Affiliations:** 1 Instituto de Medicina Molecular, Faculdade de Medicina da Universidade de Lisboa, Lisbon, Portugal; 2 Proteomics Center, Erasmus University Medical Center, Rotterdam, The Netherlands; 3 Department of Physiology and Biophysics, University of Illinois at Chicago, Chicago, Illinois, United States of America; University College London, United Kingdom

## Abstract

Vascular Endothelial Growth Factor Receptor-2 (VEGFR2) is the major mediator of the angiogenic effects of VEGF. In addition to its well known role as a membrane receptor that activates multiple signaling pathways, VEGFR2 also has a nuclear localization. However, what VEGFR2 does in the nucleus is still unknown. In the present report we show that, in endothelial cells, nuclear VEGFR2 interacts with several nuclear proteins, including the Sp1, a transcription factor that has been implicated in the regulation of genes needed for angiogenesis. By *in vivo* chromatin immunoprecipitation (ChIP) assays, we found that VEGFR2 binds to the Sp1-responsive region of the *VEGFR2* proximal promoter. These results were confirmed by EMSA assays, using the same region of the *VEGFR2* promoter. Importantly, we show that the VEGFR2 DNA binding is directly linked to the transcriptional activation of the *VEGFR2* promoter. By reporter assays, we found that the region between -300/-116 relative to the transcription start site is essential to confer VEGFR2-dependent transcriptional activity. It was previously described that nuclear translocation of the VEGFR2 is dependent on its activation by VEGF. In agreement, we observed that the binding of VEGFR2 to DNA requires VEGF activation, being blocked by Bevacizumab and Sunitinib, two anti-angiogenic agents that inhibit VEGFR2 activation. Our findings demonstrate a new mechanism by which VEGFR2 activates its own promoter that could be involved in amplifying the angiogenic response.

## Introduction

Angiogenesis is the formation of new blood vessels from a pre-existing vascular net. This process is essential during embryonic development and for normal homeostasis of adult tissues. In addition, angiogenesis was recognized to be fundamental in the progression of many pathological diseases such as cancer because it is an essential event in tumor growth and metastatic dissemination [1].

Angiogenesis is a complex dynamic process regulated by a balance between pro-angiogenic and anti-angiogenic factors. Vascular Endothelial Growth Factor (VEGF) is one of the most important pro-angiogenic factors. VEGF stimulates angiogenesis by binding to the VEGF receptor (VEGFR)-1 and VEGFR2 receptor tyrosine kinases (RTKs) on the cell surface of endothelial cells (EC) [2]. Both VEGFR1 and VEGFR2 have seven Ig-like-domains in the extracellular domain, a single transmembrane region and a split tyrosine kinase intracellular domain [2]. VEGFR2 is considered to be the major mediator of several physiological and pathological effects of VEGF on EC. These include proliferation, survival, migration and permeability [2]. VEGF binds to the extracellular domain of VEGFR2 inducing receptor dimerization and autophosphorylation of specific intracellular tyrosine residues leading to the activation of different signaling pathways [2].

Recognition of the VEGF pathway as a key regulator of angiogenesis has led to the development of several VEGF-targeted agents demonstrating therapeutic efficacy in several human cancers [3]. Therefore, several approaches have been developed to inhibit VEGF signaling, including neutralization of the ligand or receptor by antibodies, and blocking VEGF receptor activation and signaling with tyrosine kinase inhibitors [4]. The pioneers of the clinical proof-of-concept for angiogenesis inhibitors are Bevacizumab (Avastin®, Genentech/Roche), a ligand-trapping monoclonal antibody [5], and Sunitinib (Sutent®, Pfizer), which targets receptor tyrosine kinases [6], principally VEGFR2. Their goal is to block the VEGF signaling mediated by the plasma membrane receptor VEGFR2.

Besides the membrane localization of VEGFR2, it was demonstrated that it could also be found in the cell nucleus. In contrast to VEGFR1, we found that VEGFR2 translocates to the nucleus upon VEGF stimulation in a process that required phosphorylation of the receptor [7]. Furthermore, we demonstrated that *in vitro* wounding of ECs monolayers leads to a rapid and transient internalization of VEGF and VEGFR2 to the nucleus, which is essential for monolayer recovery [7]. In proliferative tumor and leukemia cells, it was also found that phosphorylated VEGFR2 has a nuclear expression [8], [9], [10], [11], [12], suggesting that molecular mechanisms that contribute to tumor angiogenesis might require a specific activity of this protein in the nucleus. Taken together, these studies document the presence of VEGFR2 in the nucleus and suggest that nuclear VEGFR2 might amplify the angiogenic response. However, the precise activity of VEGFR2 in the nucleus is unknown.

Here, we demonstrate a previously unrecognized function for nuclear VEGFR2 as a putative transcription factor that is involved in the regulation of its own transcription. We show that VEGFR2 binds to and activates its own promoter *in vivo* in VEGF-activated EC. Moreover, we observed that this mechanism is blocked by treating EC with Bevacizumab or Sutent.

## Results

### VEGFR2 nuclear internalization is a dynamic process in EC

**Figure 1:**
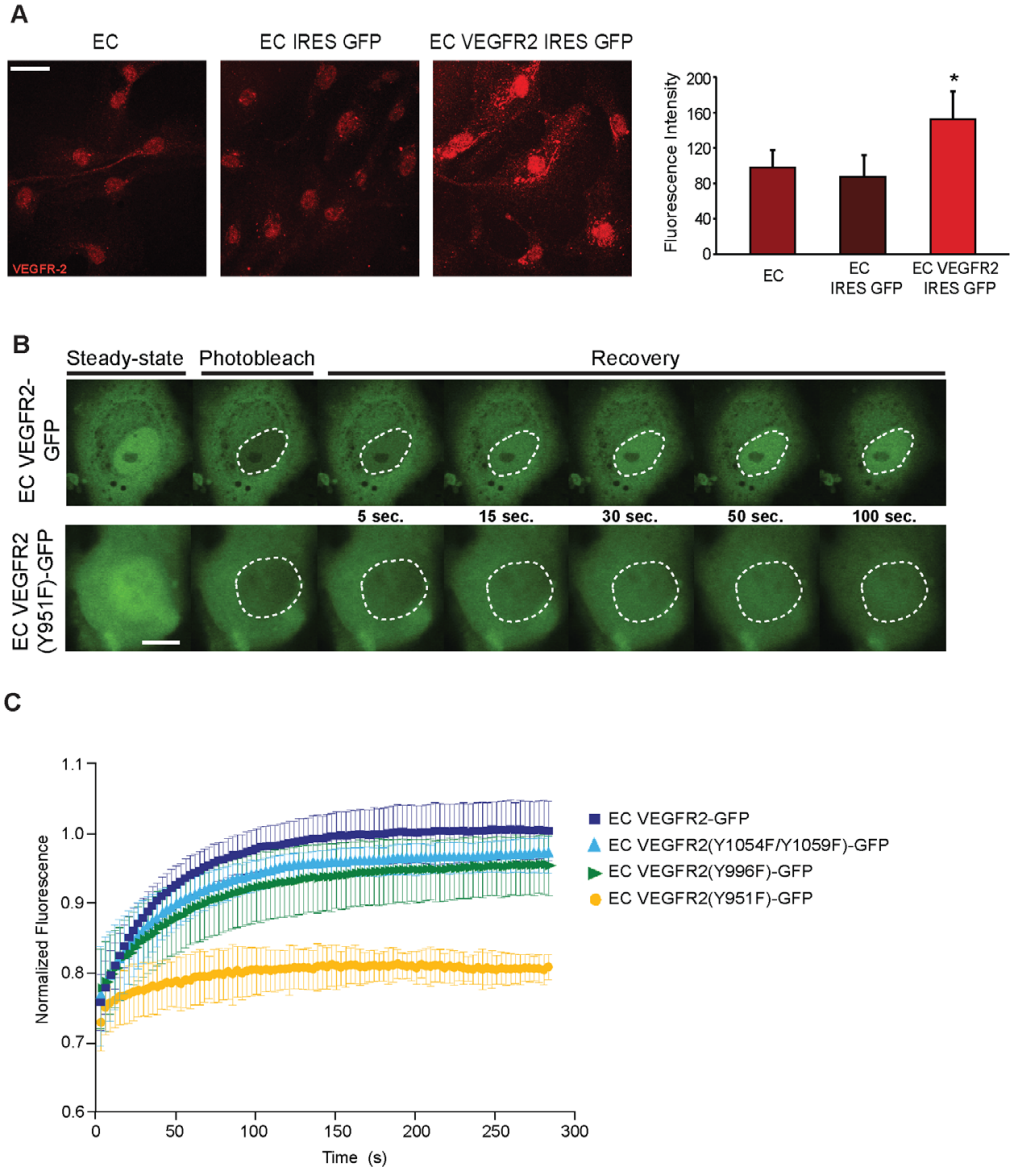
VEGFR2 nuclear translocation is a rapid process that is affected by the VEGFR2 tyrosine 951. (A) EC, EC IRES GFP and EC VEGFR2 IRES GFP were cultured in growing media and VEGFR2 overexpression was analysed by immunofluorescence. Cells were stained with a rabbit anti-human VEGFR2 antibody (Alexa 594). Results shown are representative z-projections of at least three independent experiments. Scale bar: 20 µm. Right panel shows mean fluorescence intensity of VEGFR2 in the cell nucleus. *p<0.0001. (B and C) FRAP analysis was performed in EC VEGFR2-GFP and mutants EC VEGFR2(Y1054F/Y1059F)-GFP, EC VEGFR2(Y996F)-GFP and EC VEGFR2(Y951F)-GFP. Fluorescence signal of the entire nucleus was photobleached with a single 488-nm high intensity laser pulse and subsequent fluorescence recovery was recorded for 280 s. (B) Selected images of VEGFR2-GFP protein in EC VEGFR2-GFP (upper panel) and EC VEGFR2(Y951F)-GFP (lower panel) before bleaching (steady-state) at the indicated intervals post-bleaching (from 5 to 100 s). White circles indicate the bleached region. (C) Fluorescence intensity in the bleached region was measured every 5 s for 280 s and normalized for the initial intensity. Data show results obtained in three independent experiments, with at least ten different cells analysed in each case. Error bars represent standard deviation (SD).

**Figure 2:**
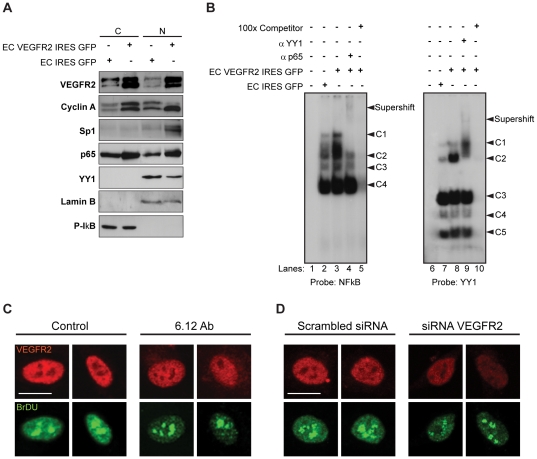
VEGFR2 nuclear internalization levels correlate with transcriptional activity of EC. (A) Cytoplasmic (C) and nuclear extracts (N) from EC IRES GFP and EC VEGFR2 IRES GFP were analysed by Immunoblot with antibodies against VEGFR2, Cyclin A, Sp1, p65, YY1. P-IkB and Lamin B were used as cytoplasmic and nuclear controls, respectively. (B) Nuclear extracts from EC IRES GFP (lanes 2,7) and EC VEGFR2 IRES GFP (lanes 3,8) were incubated with NFkB (left panel, lanes 2 and 3) or YY1 (right panel, lanes 7 and 8) radiolabeled probes. Four NFkB (C1–C4) or five YY1 complexes (C1–C5) are indicated with black arrows. Specific anti-p65 (lane 4) or anti-YY1 (lane 9) were introduced in the binding reaction to analyse the appearance of a supershift complex (as indicated in both panels) in EC VEGFR2 IRES GFP cells. Using the same cells, a competitive assay using 100x excess of cold probe of NFkB (lane 5) or YY1 (lane10) was performed. Control lanes 1 and 6 contain only the radiolabeled probes. (C) EC were cultured in growing media, treated or not with 6.12 Ab for 1 h and incubated with 5-FU for 15 min. (D) EC were cultured in growing media and transfected with scrambled siRNA or VEGFR2 siRNA. EC were incubated with 5-FU for 15 min. (C and D) 24 post-transfection. Cells were fixed and sequentially labeled on the same slide with a rabbit anti-human VEGFR2 (red fluorescence) and a mouse anti-human BrdU antibody (green fluorescence) and analysed by confocal microscopy. Results shown are representative z-projections of three independent experiments. Scale bar: 20 µm.

In order to investigate the possible functions of nuclear VEGFR2 we established an EC model of VEGFR2 over expression (EC VEGFR2 IRES GFP) using a lentiviral infection approach. Positively transduced cells were sorted using GFP expression and VEGFR2 over expression was confirmed by both immunofluorescence and confocal microscopy (Figure 1A) and immunoblot analysis (Figure 2A, first panel). By confocal analysis we found that VEGFR2 expression was increased both in the cytoplasm and nucleus of EC VEGFR2 IRES GFP, when compared to untransduced EC or EC transduced with a control vector (EC IRES GFP), (Figure 1A). Our data suggest that EC over expressing VEGFR2 results in high levels of this protein that are not degraded and also accumulate in the nucleus.

We decided to further investigate the nuclear internalization of VEGFR2 by performing photobleaching studies on living EC to measure the nuclear turnover of GFP tagged VEGFR2 (Figure 1B, C). First, we photobleached VEGFR2-GFP in the whole cell nucleus and then quantified the nuclear fluorescence recovery by time-lapse imaging. According to our results, a full recovery of the fluorescence signal was observed within 100 s (Figure 1B, upper panel, and C), suggesting a rapid turnover of VEGFR2-GFP between the cytoplasm and the nucleus. By performing studies with several deletion mutants we have previously found that the tyrosine residues present in the C-terminal tail do not change the VEGFR2 nuclear localization, in contrast to others such as 951, 996, 1053 and 1059 [7]. According to these results, we constructed tyrosine to phenylalanine VEGFR2 point mutants followed by photobleaching studies. Interestingly, we found that a single point mutation at tyrosine 951 of the VEGFR2 results in a slower turnover rate compared to EC expressing the wild-type protein, with fluorescence intensity not recovering to its pre-photobleaching baseline within the duration of the FRAP experiment (Figure 1B, lower panel, and C). EC expressing mutations in other tyrosine residues (such as Y1059, Y1054 and Y996) were evaluated and presented similar recovery kinetics following bleaching when compared to VEGFR2-GFP cells (Figure 1C).

Taken together, our results suggest that the translocation of VEGFR2 from the cytoplasm to the nucleus is a rapid and dynamic process in which the tyrosine residue 951 plays an important role.

### VEGFR2 nuclear internalization is correlated with transcriptional activity in EC

We tested if increased levels of VEGFR2 modified the levels of nuclear proteins involved in cell proliferation and survival processes that are also involved in angiogenic responses: Cyclin A [13], [14], p65 (NFkB), [15], [16], [17], Sp1 [18], [19] and YY1 [20], [21], [22]. We observed increased levels of Cyclin A, p65 (NFkB) and Sp1 in the nucleus of EC VEGFR2 IRES GFP, compared to control EC IRES GFP (Figure 2A). The expression of YY1 was not significantly altered in the same cells (Figure 2A). Since some of these nuclear proteins are transcription factors (TFs), we evaluated whether their nuclear expression levels were mirrored by a change in their DNA binding activities using EMSA assays. We found a p65 (NFkB) increased DNA binding activity in the VEGFR2 over expressing cells, compared to control EC IRES GFP (Figure 2B, left panel), which is consistent with the increased p65 protein levels in the nucleus (Figure 2A, right panel). Interestingly, the DNA binding activity of YY1 was also increased in EC over expressing VEGFR2 (Figure 2B). These results indicate that binding activities of several TFs are increased in EC expressing VEGFR2, suggesting an enhanced transcriptional activity in these cells. For that reason, we decided to test if the levels of transcription in EC were also altered when the nuclear accumulation of VEGFR2 was experimentally reduced. For this, we took advantage of our previous observation that a neutralizing antibody against VEGFR1 (6.12 Ab) decreases VEGFR2 levels in the nucleus [7]. Using a 5-fluorouracil (5-FU) incorporation assay, we observed that the levels of transcription were decreased after 6.12 Ab treatment, compared to control cells (Figure 2C). Similar results were obtained when the levels of VEGFR2 were reduced by using the siRNA technology (Figure 2D). As shown in Figure S1, a pool of VEGFR2 siRNA oligos used in our experiments effectively abrogated the VEGFR2 expression as assessed by qRT-PCR (by 70% compared with the scrambled siRNA oligos).

Taken together, these results suggest that increased VEGFR2 accumulation in the nucleus is correlated with increased transcription.

### Nuclear VEGFR2 interacts with the transcription factor Sp1 in the nucleus of EC

**Figure 3:**
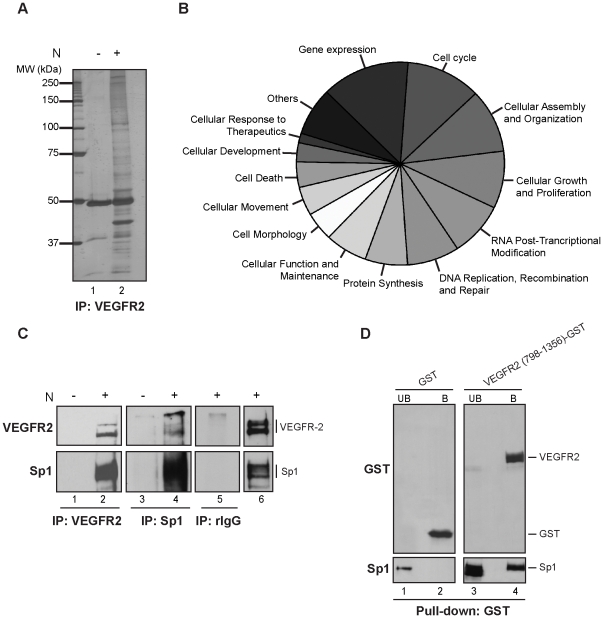
Nuclear VEGFR2 interacts with the transcription factor Sp1 in the nucleus of EC. (A) Immunoprecipitation (IP) of 1 mg EC nuclear extract with anti-human VEGFR2 was performed and resolved in 8% SDS-PAGE, following silver staining (lane 2). VEGFR2 antibody plus beads (without N) were used as negative control for immunoprecipitation (lane 1). The protein marker is shown as molecular weight (MW) in thousands. (B) Representation of the mass spectrometry analysis of the nuclear VEGFR2 IP, showing the categories for the different biological functions of the identified proteins (p<0.05). (C) Immunoprecipitation (IP) of EC nuclear extracts (N) was conducted with the VEGFR2 (lane 2), Sp1 (lane 4) and rabbit IgG (rIgG-lane 5) antibodies followed by VEGFR2 (upper panel) or Sp1 (lower panel) immunoblotting. VEGFR2 (lane 1) or Sp1 (lane 3) antibodies plus beads (without N) were used as negative controls for immunoprecipitation. Non-immunoprecipitated nuclear cell extract (lane 6) was also included in the experiment. (D) Pull-down assay: Sp1 protein fused to a HA tag was incubated with GST alone (lanes 1 and 2) or VEGFR2 (789-1356)-GST (lanes 3 and 4). GST-unbound (UB) (lanes 1 and 3) and bound (B) fractions (lanes 2 and 4) were loaded and analyzed with GST (upper panel) and Sp1 (lower panel) antibodies.

The above results suggest that the nuclear levels of VEGFR2 could modulate cell transcription and we decided to investigate the specific role of VEGFR2 in this process. We asked if VEGFR2 interacts with nuclear proteins and if it modulates cell transcription. To address this question, VEGFR2 was immunoprecipitated from EC nuclear extracts and the proteins that directly or indirectly interacted with VEGFR2 were identified by Mass Spectrometry (MS) analysis (Figure 3A,B). Approximately 310 proteins were identified with high confidence with the Mascot algorithm. None of these proteins have an exclusively cytoplasmic or nuclear localization. Proteins with a Mascot score greater than 200 were listed in Table S1. Analysis of these possible partners with Ingenuity Pathway Analysis (IPA) identified 95 proteins in 22 enriched categories for biological functions as represented in Figure 3B. Interestingly, proteins involved in gene expression are among the most abundant, suggesting that nuclear VEGFR2 could interact with proteins involved in gene transcription.

In order to confirm the data obtained by MS, we performed immunoblot on nuclear VEGFR2 immunoprecipitates. We confirmed an interaction between VEGFR2 and Sp1 (Figure 3C). Interestingly, Sp1 is a transcription factor that regulates multiple genes important to angiogenesis. The antibody against Sp1 does not cross-react with other members of the Sp family, indicating a specific interaction between VEGFR2 and Sp1. Furthermore, we performed pull-down assays using purified proteins and our results suggest an interaction between Sp1 and the region containing amino acids 789 to 1356 of VEGFR2 (Figure 3D).

### Nuclear VEGFR2 binds to and activates the *VEGFR2* proximal promoter in EC

**Figure 4:**
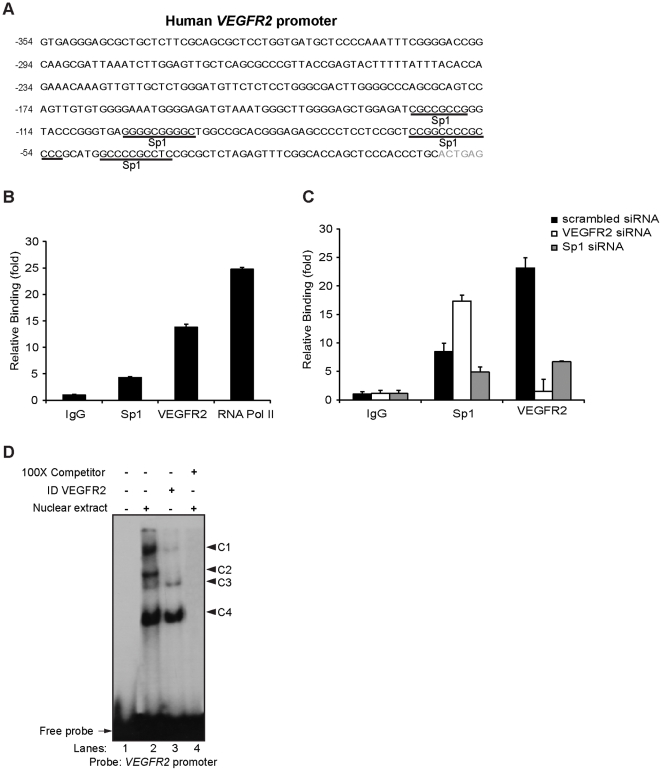
Nuclear VEGFR2 binds to the *VEGFR2* proximal promoter in EC. (A) Sequence of the human *VEGFR2* proximal promoter (retrieved from Ensemble database accession number: ENSG00000128052) and outline of putative Sp1 binding sites. The transcription start site is indicated in gray. (B) ChIP assays of the *VEGFR2* proximal promoter were performed using EC cultured in growing media. Antibodies against VEGFR2 and Sp1 were used. Normal rabbit/mouse IgG were used as control. Also, an antibody for RNA Pol II was used to test the promoter activity. All values are relative to control IgG background and normalized to an intergenic region. Data are mean ± s.e.m. of triplicates and represents three independent experiments. (C) ChIP assays of the *VEGFR2* proximal promoter were performed in EC 24 h post-transfection of scrambled siRNA or VEGFR2 siRNA or Sp1 siRNA. Antibodies against VEGFR2 and Sp1 were used. Normal rabbit IgG was used as control. Values are relative to control IgG background and normalized to an intergenic region. Data are mean ± s.e.m. of triplicates and represents three independent experiments. (D) EMSA analysis of the *VEGFR2* promoter with EC nuclear extracts (lane 2) or VEGFR2 immunodepleted (ID VEGFR2) extract (lane 3) were conducted. Four complexes (C1-C4) are indicated with black arrows. A competitive assay using 100x excess of cold probe of VEGFR2 promoter was conducted (lane 4) using EC nuclear extract. Control lane 1 contains only the radiolabeled probe.

Since it was previously shown that Sp1 is involved in the transcriptional regulation of *VEGFR2* gene [23], [24] and because our results suggest a nuclear interaction between Sp1 and VEGFR2 (Figure 3C), we hypothesized that VEGFR2 could be involved in the regulation of its own transcription. In order to answer to this question, we decided to investigate if VEGFR2 could bind to its own promoter. Quantitative ChIP assays were performed on EC, cultured in growing media. We chose a region of the human *VEGFR2* proximal promoter that comprises five Sp1 binding sites between -300/+1 relative to the transcription start site (Figure 4A). We observed that Sp1 binds to the *VEGFR2* proximal promoter (4.2 fold ±0.14 increase relative to the control IgG), (Figure 4B), which was consistent with previous reports [23], [25]. Interestingly, we observed a 13.8 fold ±0.55 increase in binding of VEGFR2 relative to the negative control IgG (Figure 4B). As expected, when EC are transfected with Sp1 siRNA in order to reduce its expression, the binding of Sp1 to its promoter is significantly decreased (Figure 4C). Moreover, the binding of VEGFR2 to its own promoter is abrogated in EC transfected with VEGFR2 siRNA (Figure 4C). Note that in both transfections, the expression of *Sp1* and *VEGFR2* were downregulated approximately 70% compared with that of scrambled siRNA-transfected EC (Figure S1). Curiously, we also found that the binding of Sp1 to *VEGFR2* promoter is significantly increased when the expression of VEGFR2 is downregulated by siRNA. In contrast, the binding of VEGFR2 to its own promoter is reduced in EC transfected with Sp1 siRNA (Figure 4C).

In order to confirm that VEGFR2 binds to its own promoter, we performed EMSA assays using as a probe the same region of the *VEGFR2* proximal promoter analyzed by ChIP. We identified four complexes (C1–C4) with distinct electrophoretic mobilities (Figure 4D, lane 2) which did not form when an excess of cold probe was introduced in the reaction (Figure 4D, lane 4), establishing their specificity. To evaluate the presence of VEGFR2 in the shifted complexes, we first tried a supershift assay using an antibody against VEGFR2, which failed to produce any change in the mobility of the shifted complexes (data not shown). As the VEGFR2 antibodies were active in immunoprecipitation experiments, we used an immunodepletion approach to evaluate the presence of VEGFR2 in the shifted complexes. Using these VEGFR2-immunodepleted nuclear extracts in the EMSA assays we observed an absence of the C2 complex and a strong reduction in the intensity of the C1 complex while the C3 and C4 complexes were not significantly altered (Figure 4D, lane 3). An IgG-depleted control extract did not affect the intensity of these complexes (Figure S2B). Simultaneously, the VEGFR2 depletion in the protein extracts was confirmed by immunoblot (Figure S2A). These results are consistent with the presence of VEGFR2 in the C1 and C2 complexes. A similar experiment performed using Sp1-depleted extract showed a decrease in the intensity of C1 and C2 complexes (Figure S2B), suggesting that Sp1 and VEGFR2 are present in the same DNA/protein complexes.

**Figure 5:**
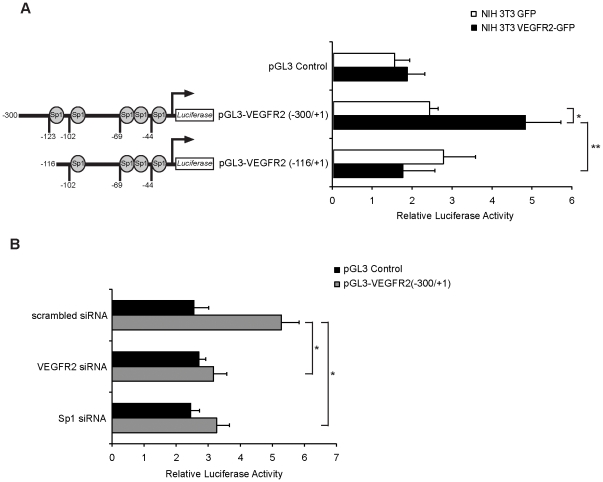
Nuclear VEGFR2 activates the human *VEGFR2* proximal promoter. (A) NIH 3T3 GFP and NIH 3T3 VEGFR2-GFP were transfected with pGL3 control or pGL3 VEGFR2 (-300/+1) or pGL3 VEGFR2 (-116/+1). The β-gal plasmid was co-transfected as a control. Promoter activities were measured with luciferase activity normalized to β-gal. The results are expressed as the relative luciferase activities. Data are mean ± s.e.m. of relative luciferase activities from four independent experiments, each performed in triplicate. *p = 0.007; **p = 0.001). (B) NIH 3T3 VEGFR2-GFP were transfected with scrambled siRNA, VEGFR2 siRNA or Sp1 siRNA. At 48 h post-transfection the relative luciferase activity of pGL3 control or pGL3 VEGFR2 (-300/+1) was measured. Data are mean ± s.e.m. of relative luciferase activities from three independent experiments, each performed in triplicate, (*p  = 0.003).

Finally, we investigated the ability of the nuclear VEGFR2 to transcriptionally activate its own promoter using luciferase reporter assays. For these experiments we used the 3T3 VEGFR2-GFP cells, which constitutively express VEGFR2, and compared to control 3T3 GFP cells that do not express VEGFR2 [7]. Transfection of a pGL3 control vector alone did not produce significant differences in luciferase activities in both cell lines (Figure 5A). However, when we transfected a construct including the *VEGFR2* proximal promoter spanning -300/+1, we observed a significantly higher luciferase activity in the VEGFR2-GFP cells (4.8 fold ±0.88, p = 0.007) compared to control cells (2.4 fold ±0.22), (Figure 5A). In accordance, we confirmed a reduction in luciferase activity in VEGFR2-GFP cells cotransfected with VEGFR2 siRNA when compared to scrambled siRNA-cotransfected cells (Figure 5B). Moreover, no increased luciferase activity over basal levels (1.74 fold ±0.8) was observed in the 3T3 VEGFR2-GFP cells, when transfected with a reporter construct containing a shorter fragment (-116/+1) of the *VEGFR2* promoter (Figure 5A). These results suggest that VEGFR2 is indeed able to activate transcription from its own promoter and that this activation requires the region between -300/-116 relative to the transcription start site. Consistent with the observed decrease in VEGFR2 binding to its own promoter when the expression of Sp1 is reduced by siRNA (Figure 3C), we also observed a significant decrease of the luciferase activity in VEGFR2-GFP cells cotransfected with Sp1 siRNA (Figure 5B).

Taken together, these data strongly suggest a previously unrecognized function of nuclear VEGFR2 as a possible transcription factor involved in the regulation of its own transcription.

### VEGFR2 binding to its own promoter is dependent of VEGFR2 activation

**Figure 6:**
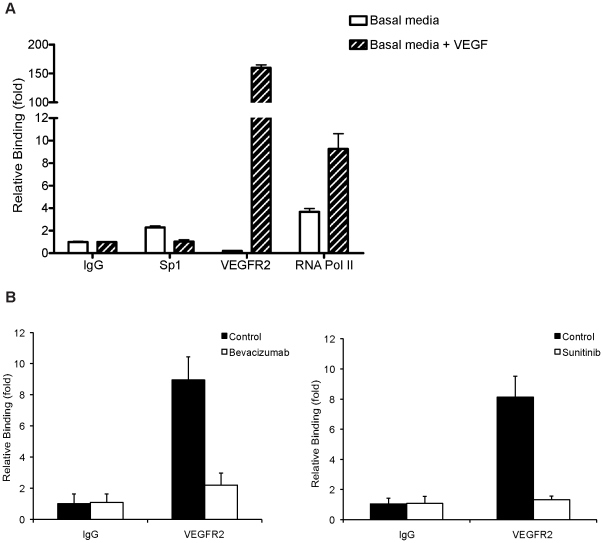
VEGFR2 binding to its own promoter is dependent of VEGFR2 activation. ChIP assays of the *VEGFR2* proximal promoter were performed using (A) EC cultured in basal medium for 48 h, and stimulated or not with VEGF (20 ng/ml) for 30 min. Antibodies against VEGFR2 and Sp1 were used. Normal rabbit/mouse IgG were used as control. Also, an antibody for RNA Pol II was used to test the promoter activity. Values are relative to control IgG background and normalized to an intergenic region. Data are mean ± s.e.m. of triplicates and represents three independent experiments. (B) EC cultured in growing media were treated or not with 0.5 mg/ml Bevacizumab (left panel) or 0.1 µM Sunitinib (right panel) for 16 h. In the Sunitinib experiments, DMSO was used as vehicle. ChIP values are relative to control IgG and normalized to an intergenic region. Data are mean ± s.e.m. of triplicates and represents three independent experiments.

To further analyze the possible functional relevance of the VEGFR2 binding to its own promoter, we took advantage of our finding that VEGFR2 nuclear translocation requires activation by VEGF [7]. We did not observe DNA binding of VEGFR2 (0.2 fold ±0.03) when EC were cultured under basal medium (without supplements or serum), which is consistent with the absence of nuclear VEGFR2 in these culture conditions [7]. Also, the Sp1 binding was negligible under these conditions (2.3 fold ±0.13), (Figure 6A). However, after 30 min of VEGF stimulation, we observed a strong increase in binding of VEGFR2 (159.6 fold ±5.21) to its own promoter (Figure 6A). In the same cells, we failed to observe an increment of Sp1 binding (1.03 fold ±0.17, relative to control IgG), (Figure 6A). These results demonstrate that VEGFR2 activation by VEGF is crucial for VEGFR2 binding to its own promoter *in vivo,* suggesting that VEGFR2, as a nuclear protein, could be involved in amplifying the angiogenic response. To further explore this idea, we treated the EC with two anti-angiogenic agents, which block the VEGFR2 activation, in order to evaluate their effect on VEGFR2 binding to its own promoter. Our results showed that both Bevacizumab, a monoclonal antibody against VEGF and Sunitinib which inhibits VEGFR, PDGFR and c-KIT signaling, led to a strong reduction of the binding of VEGFR2 to its own promoter, as estimated by quantitative ChIP (Figure 6B). As a control, ECs were also treated with Iressa, an inhibitor of epidermal growth factor receptor (EGFR) that has no effect on VEGFR2 activation. Our results (Figure S3) show that Iressa has no effects on VEGFR2 binding to its own promoter.

Taken together, our results suggest that anti-angiogenic agents that block VEGFR2 activation significantly decrease the level of VEGFR2 DNA binding activity.

## Discussion

The classical view of signaling through VEGFR2 considers that the membrane receptor is phosphorylated upon ligand binding, activating intracellular signaling cascades that regulate a wide range of biological outcomes, including cellular survival, proliferation, migration and differentiation [2]. Until recently, internalization of VEGFR2 was thought to be the pathway for downregulation of the signaling through receptor degradation. Consistent with this, it was found that VEGFR2 is ubiquitylated by c-Cbl [26] and that activated PKC marks the receptor for internalization and degradation [27]. However, increasing evidence indicates that internalized VEGFR2 may also have signaling activity. For instance, it has been shown that phosphorylated VEGFR2 can be internalized in a VE-cadherin-dependent manner to endosomal compartments, retaining activation of signaling pathways and sustaining cell proliferation and migration, rather than receptor degradation [28]. Recently, it was shown that ephrin-B2 activation controls VEGFR2 internalization, which is required for activation and downstream signaling of the receptor during vascular sprouting in physiological and pathological conditions [29], [30]. We also demonstrated that the nuclear internalization of VEGFR2 is required for endothelial recovery following injury [7]. Finally, we and others found constitutive nuclear localization of VEGFR2 in proliferative tumor cells, suggesting that this protein may be involved in nuclear molecular mechanisms that contribute to tumor progression [8], [9], [10], [11], [12]. Taken together, these different studies suggest that the intracellular trafficking of VEGFR2 is linked to its signaling activity that contributes to the amplification of the angiogenic response.

The mechanism by which VEGFR2 translocates to the nucleus is not yet completely understood. VEGFR2 may be internalized preferentially via a caveolar pathway and transported to perinuclear caveosomes [31], [32], [33], [34]. In support of this, we and others found that VEGFR2 colocalized with caveolin-1 [7], [35]. It is known that caveolae are transported from the membrane to intracellular organelles along microtubules [36] and dynamin-2, a well-established regulator of caveolar endocytosis, seems to require interactions with functional microtubules to stimulate its GTPase activity and promote vesicular transport [37], [38], [39], [40]. This is consistent with our finding that drugs inhibiting microtubule polymerization blocked the internalization of VEGF-VEGFR2 complex [7]. It was also reported that inhibitors of dynamin-2 blocked the VEGF-induced internalization of VEGFR2, resulting in decreased tip EC filopodia extensions [29]. From caveosomes, internalized cargo may be delivered to the endoplasmic reticulum providing a transport pathway to the nucleus. Our previous findings [7], suggest that VEGFR2 nuclear internalization requires the nuclear pore complex (NPC) since an accumulation of VEGFR2 is observed after treating EC with an inhibitor of the NPC. The typical mechanism for the import through the NPC is mediated by the binding of the protein nuclear localization signals (NLS) to importin α and β to form a complex that interacts with NPC so that the protein can enter the nucleoplasm [41]. However, the presence of NLS in VEGFR2 sequence was neither described nor identified in the bioinformatics analyses performed by us. Therefore, we hypothesize that the model proposed for FGFR1 internalization could also apply to VEGFR2. FGFR1, which is also devoid of a NLS, is chaperoned to the nucleus by its ligand FGF2. The binding of FGF2, which harbors NLS sequences, results in the nuclear translocation of the receptor-ligand complex in an importin α-dependent manner [42]. VEGF has five potential NLS sequences in the C-terminal region [43] and it is possible that they drive the complex VEGF-VEGFR2 through the NPC to the nucleus.

In the present report we have shown in living EC that VEGFR2 rapidly translocates to the nucleus and the VEGFR2 tyrosine residue 951 plays an important role in this dynamic process. The role that VEGFR2 might play in the nucleus has remained undisclosed. Here, we show for the first time to our knowledge that nuclear VEGFR2 has transcriptional activity. In particular, we show that VEGFR2 binds to its own promoter in VEGFR2-activated EC *in vivo* and that VEGFR2 can activate transcription from this promoter in reporter assays. These findings suggest that VEGFR2 might participate in the positive feedback regulation of its own expression. This is consistent with previous reports showing that VEGF binding to membrane VEGFR2 results in increased levels of *VEGFR2* gene transcription and protein expression [44]. Similarly, it was observed that mechanoactivation produces translocation of VEGFR2 to the nucleus [45], which is accompanied by an up-regulation of the *VEGFR2* gene transcription [46].

Our results now indicate that this increase in VEGFR2 expression depends, at least in part on VEGFR2 transcriptional activity. Our EMSA data revealed the existence of different VEGFR2 containing complexes (C1 and C2 complexes) with different mobilities when bound to the promoter, suggesting that VEGFR2 interacts with additional molecules when bound to DNA. Our MS profiling data seems to support this idea. In particular, Sp1 stands out as one of the VEGFR2-interacting proteins in the nucleus of EC. This is consistent with previous data showing that Sp1 is implicated in the transcriptional regulation of genes important to angiogenesis, including, *VEGF* and *VEGFR2* [23], [24], [47]. Co-immunoprecipitation experiments showed that Sp1 and VEGFR2 interact. Pull-down assay experiments using purified proteins further confirmed that the interaction between VEGFR2 and Sp1 is direct. Our EMSA data using Sp1 or VEGFR2 depleted extracts also indicate that Sp1 and VEGFR2 are present in the same protein-DNA complexes. Interestingly, the -300/+1 bp region of the *VEGFR2* promoter, identified as one of the key elements for the regulation of *VEGFR2* expression [24], [25], [48], [49], contains five Sp1 binding sites and is able to bind both Sp1 and VEGFR2. Our reporter assays show that the region between -300/-116 relative to transcription start site is essential to confer the *VEGFR2* promoter VEGFR2-dependent transcriptional activity. Moreover, our results indicate that VEGFR2 is essential for the activity of its own promoter since cells that do not express VEGFR2 have significantly lower levels of the *VEGFR2* promoter activity compared to VEGFR2-expressing cells. Accordingly, the levels of the *VEGFR2* promoter activity observed in VEGFR2-expressing cells are significantly decreased if the expression of VEGFR2 is reduced by siRNA. A definitive proof of VEGFR2 transcriptional activity would require a direct analysis of the endogenous *VEGFR2* locus. This could be overcome by generating EC carrying a reporter gene (e.g GFP) knocked-in into the *VEGFR2* locus. A recently developed technology, which allows homologous recombination in somatic cells using recombinant Adeno-Associated Virus (rAAV), might facilitate the establishment of such a reporter line [50], [51], [52]. In this system, the reduction of the *GFP* reporter gene transcripts following VEGFR2 siRNA experiments would confirm the VEGFR2-mediated transcriptional activity on the endogenous locus.

However, at the moment we do not know if VEGFR2 binds to a consensus DNA sequence and the nature of this sequence. Clearly, identification of other transcriptional targets of VEGFR2 will help to address this issue. Also, understanding the mechanism of VEGFR2 transcriptional activity will require the complete identification of the molecules interacting with VEGFR2 at the promoters/enhancers. In EC, binding of VEGFR2 to DNA requires VEGF-activation, since this binding cannot be detected in EC cultured in the absence of this growth factor. Moreover, EC treated with anti-angiogenic agents that block VEGFR2 activation present negligible levels of VEGFR2 DNA binding activity. This is consistent with the finding that the nuclear translocation of the receptor is dependent of VEGF activation [7], and further supports the idea that nuclear translocation/transcriptional activity of VEGFR2 is an integral part of the signaling mediated by this receptor.

While in EC VEGFR2 nuclear translocation and consequently its DNA binding depends on VEGFR2 activation by VEGF, it has been reported that some tumor cells present constitutive nuclear localization of VEGFR2. If these tumor cells also present constitutive VEGFR2 transcriptional activity, this could be an additional mechanism that plays a role in tumor angiogenesis. Therefore, the analysis of VEGFR2 transcriptional activity in those tumors and the identification of the target genes will surely help to better understand its putative role in tumor angiogenesis and to devise novel therapeutic approaches.

In conclusion, our findings provide novel insights into the role of VEGFR2 as a nuclear protein. Here, we demonstrate that in VEGF-activated EC, nuclear VEGFR2 may act as a transcription factor by binding to and activating its own promoter. By this mechanism nuclear VEGFR2 could be involved in amplifying the angiogenic response.

## Materials and Methods

### Reagents

VEGF (20 ng/ml) was purchased from Sigma-Aldrich, USA. Bevacizumab (Avastin®, Genentech/ Roche, USA), (0.5 mg/ml) was provided by the Oncology Service of Santa Maria Hospital. Sunitinib (0.1 µM) was provided by Pfizer International, USA. Iressa (0.1 µM) was purchased from Tocris Bioscience, UK. 5-fluorouracil (5-FU, 2 mM) was purchased from Sigma-Aldrich, USA.

### Cell culture

Primary Human Umbilical Vein Endothelial Cells (EC) were kindly provided by Dr Shahin Rafii (Cornell University Medical College, New York, USA). EC, passage 4–8, were cultured in 0.02% gelatin-coated dishes in growing endothelial medium (basal EBM-2 medium supplemented with EGM-2 singlequots, BBE and 5% of Fetal Bovine Serum (FBS)) as provided by the manufacturers (Lonza, USA). In basal media experiments, upon reaching confluence EC were cultured in basal EBM-2 medium for 48 h. HEK-293 T cells were cultured in DMEM (Invitrogen Corporation, USA) supplemented with 10% FBS. NIH 3T3 GFP and NIH 3T3 VEGFR2-GFP cells were described and characterized in [7] and were cultured in DMEM supplemented with 10% FBS and 800 µg/ml Neomycin-G418 (Invitrogen Corporation, USA).

### Construction of the VEGFR2 point mutants by site directed-mutagenesis

Tyrosine to phenylalanine VEGFR2 point mutants were generated by site-directed mutagenesis using the overlap extension method. Two separate amplification reactions were first performed using the pEGFP-VEGFR2 as template; one using the primer A: 5′ C GTC ATG GAT CCA GAT GAA CTC C 3′ (sense) and the mutated antisense primer (listed below), the other using the mutated sense primer (listed below) and the primer B: 5′ TA G GT CAG GGT GGT CAC GAG 3′ (antisense).

The mutated primers designed to replace tyrosine (Y) to phenylalanine (F-bold) residues in Y951 were: 5′ GGG AAA GAC TTC GTT GGA GCA 3′ (sense) and 5′ TGC TCC AAC GAA GTC TTT CCC 3′ (antisense); in Y996 were 5′ T CCT GAA GAT CTG **TTT** AAG GAC TTC CTG 3′ (sense) and 5′ G GAA GTC CTT **AAA** CAG ATC TTC AG 3′ (antisense); in Y1054 were 5′ GCC CGG GAT ATT **TTT** AAA GAT CCA G 3′(sense) and 5′ TGG ATC TTT **AAA** AAT ATC CCG GGC C 3′ (antisense); in Y1059 were 5′ GAT CCA GAT **TTT** GTC AGA AAA GGA G 3′ (sense) and 5′ C TCC TTT TCT GAC **AAA** ATC TGG ATC T 3′ (antisense).

The thermal amplification conditions were 95°C/5 min, 35 cycles (95°C/1 min, 61°C/1 min, 72°C/1 min), 72°C/10 min. An overlapping reaction was performed using the mutated products from the first PCR (2–5%) and the sense A and antisense B primers. The thermal amplification conditions were 95°C/5 min, 35 cycles (95°C/1 min, 58°C/1 min, 72°C/1 min), 72°C/10 min. PCR products were inserted into the *Bam*HI/ *Apa*I sites of pEGFP-VEGFR2. All constructs were confirmed by DNA sequencing.

### Generation of Lentiviral vectors expressing VEGFR2

The VEGFR2 WT and tyrosine to phenylalanine mutants fused to GFP were released from pEGFP-VEGFR2 using the *Sal*I/ *Hpa*I restriction sites and were cloned in the lentiviral plasmid FUGW (kindly given by Dr. Pedro Simas, Instituto de Medicina Molecular, Lisbon, Portugal) in the *Bam*HI/ *Eco*RI restriction sites, using blunt-end cloning, generating FU-VEGFR2-GFP. All constructs were confirmed by DNA sequencing. The lentiviral vector FU IRES GFP was generated by replacing the GFP of FUGW with the IRES GFP from pIRES GFP (Stratagene Inc., USA). FU-VEGFR2 IRES GFP was generated by releasing full length VEGFR2 from pSP73-VEGFR2 using *Kpn*I/ *Xho*I restriction sites and cloned in FU IRES GFP using the *Bam*HI restriction site by blunt-end cloning. All constructs were confirmed by DNA sequencing.

### Lentiviral Production

Lentiviral particles were obtained with the transfection of HEK-293T cells using a standard calcium phosphate precipitation protocol. HEK-293T cells (≈50% confluent) were transfected with the lentiviral vector plasmids FUGW, FU VEGFR2-GFP (VEGFR2 WT or tyrosine to phenylalanine mutants), FU IRES GFP or FU-VEGFR2 IRES GFP together with the HIV-1 packaging vector Delta 8.9 and the VSV-g envelope glycoprotein. The viral supernatants were collected 60 h post-transfection and filtered through a 0.45 µm pore size filter. EC were seeded at 7.5×10^4^ cells (12 well plate) 24 h before transduction and then exposed to 500 µl of virus supernatant (supplemented with polybrene to a final concentration of 4 µg/ ml). 72 h post-infection the GFP positive cells were sorted by FACS Aria (Becton, Dickinson and Company, USA). EC expressing FUGW were named EC GFP; EC expressing FU-VEGFR2(WT)-GFP were named EC VEGFR2-GFP; EC expressing FU-VEGFR2(Y1054F/Y1059F)-GFP were named EC VEGFR2(Y1054F/Y1059F)-GFP; EC expressing FU-VEGFR2(Y996F)-GFP were named EC VEGFR2(Y996F)-GFP; EC expressing FU-VEGFR2(Y951F)-GFP were named EC VEGFR2(Y951F)-GFP; EC expressing FU IRES GFP were named EC IRES GFP; EC expressing FU-VEGFR2 IRES GFP were named EC VEGFR2 IRES GFP.

### Immunofluorescence and Confocal Microscopy

EC were cultured on gelatin-coated glass coverslips. The cells were fixed in 1% (v/v) formaldehyde/ PBS, for 10 min, at 4°C and washed in PBS. After permeabilization with 0.1% (v/v) Triton X-100 plus 5% (v/v) normal serum, cells were incubated in different conditions with the following antibodies: VEGFR2 (Santa Cruz Biotechnology, USA) at 4°C, overnight, followed by incubation with Alexa Fluor 594 (Molecular Probes, Invitrogen Corporation, USA) for an additional hour, at room temperature or BrdU (Sigma-Aldrich, USA), for 30 min, at room temperature, followed by incubation with Alexa Fluor 488 (Molecular Probes, Invitrogen Corporation, USA) for additional 30 min, at room temperature. The samples were mounted in Vectashield (Vector Laboratories, USA) and analyzed by confocal microscopy. Sets of optical sections of 5 µm intervals along the Z-axis (from bottom to top of cells) were acquired on a Zeiss LSM 510 META (Carl Zeiss, Germany) inverted laser scanning confocal microscope using a PlanApochromat 63x/1.4 oil immersion objective. Alexa Fluor 488 and GFP fluorescence were detected using the 488 nm line of an Ar laser (45 mW nominal output) and a BP 505–550 nm filter. Alexa Fluor 594 fluorescence was detected using a 594 nm HeNe laser (2 mW nominal output) and a LP 615 nm filter. Potential bleed-through from the different fluorophores was avoided by performing sequential multi-track/frame imaging sequences. Z-projections were obtained using ImageJ (http://rsbweb.nih.gov/ij/).

Live cell imaging was performed at 37°C and 5% CO_2_ on a Zeiss LSM 510 META (Carl Zeiss, Germany) inverted laser scanning confocal microscope equipped with a large incubator for temperature control and a stage incubator for CO_2_ supply (PeCon, Germany).

### Fluorescence Recovery After Photobleaching (FRAP)

Each FRAP analysis started with a single image scan followed by a bleach pulse at 100% laser power in a region of interest (ROI) that coincided with the cell nucleus (∼350 mm^2^ area). A series of 56 single-section images were then acquired at 5 s intervals for 280 s, with the first image being acquired 2 ms after the end of the photobleaching. Image acquisition was performed with laser power attenuated to 1% of the bleaching intensity.

Fluorescence intensity quantification was performed for each FRAP time series using ImageJ software (http://rsbweb.nih.gov.ij). The average fluorescence in the nucleus of bleached cells I(*t*) and the total cell fluorescence T(*t*) were calculated for each background-subtracted image at time *t*. FRAP curves for bleached cells were then normalized and corrected for loss of fluorescence due to imaging,
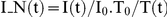
where I_0_ and T_0_ are the nuclear and total fluorescence intensities before bleaching started [53].

### 5-FU incorporation

EC were cultured in growing media on gelatin-coated glass coverslips, treated with 6.12 Ab (1 µg/ml) and scrambled or VEGFR2 siRNA following incubation with 5-FU (2 mM) for 15 min. The cells were fixed, permeabilized, and labeled sequentially for BrdU and VEGFR2 according to the immunofluorescence protocol described above.

### RNA interference

SMART pool siRNA targeting human *VEGFR2* or human *Sp1* and non-targeting pool siRNA (scrambled siRNA) were purchased from Dharmacon (UK). Knockdown of *VEGFR2* or *Sp1* was performed according to the manufacturer's recommendation. Briefly, EC were transfected with VEGFR2, Sp1 or scrambled siRNA (50 nM) using the Dharmafect 4 reagent (Dharmacon, UK). After 24 h, cells were used in qRT-PCR, ChIP, 5FU-incorporation or luciferase assays.

### Cell Fractionation and Immunoblot Analysis

Nuclear and cytoplasmic protein extracts were prepared as described [7]. Equal amounts of protein extracts were separated by SDS–PAGE, transferred to nitrocellulose and probed with antibodies against VEGFR2, P-IkB (both from Cell Signaling Technology Inc., USA), Cyclin A, Sp1, p65, YY1, Lamin B (all from Santa Cruz Biotecnology, USA).

### Immunoprecipitation

Nuclear extracts were pre-cleared with 25 µl of protein G-Sepharose beads (Sigma-Aldrich, USA). Nuclear protein supernatants were incubated with antibodies against VEGFR2 (Cell Signaling Technology Inc., USA) or Sp1 (Santa Cruz Biotecnology, USA) and rabbit control IgG (Santa Cruz Biotecnology, USA) overnight, at 4°C, and incubated with protein G-Sepharose beads for an additional hour, at 4°C. Beads were washed once in a lysis buffer containing 500 mM NaCl and twice in a lysis buffer containing 150 mM NaCl. Beads were resuspended in SDS loading buffer and boiled for 5 min. Samples were separated by SDS-PAGE followed by Immunoblot analysis or silver staining for mass spectrometry analysis.

### Pull-down assays

The GST fusion protein containing amino acids between 789-1356 of human VEGFR2 (VEGFR2 (789-1356)-GST) was purchased from Sigma-Aldrich, USA. The Sp1 protein fused to a HA tag (Sp1-HA) was obtained from Enzo Life Sciences, USA. For pull-down assays, 3 µg of Sp1-HA were incubated with glutathione-sepharose 4B beads (GE Healthcare, USA) for 1 h, at 4°C, in binding buffer containing 50 mM Tris-HCl pH 7.5, 150 mM NaCl, 10% Glicerol, 1% NP-40, 1 mM orthovanadate and complete protease inhibitors. The beads were spin down and the pre-cleared supernatant was incubated with 3 µg of purified GST or VEGFR2 (789-1356)-GST proteins overnight, at 4°C. Peptide complexes were recovered with 20 µl of glutathione-sepharose beads for 1 h, at 4°C. The supernatants were kept as the unbound fractions (UB) and the beads were washed eight times in the binding buffer. Protein were eluted from the beads in reducing laemmli's buffer, resolved by SDS-PAGE, transferred to nitrocellulose and analyzed by Immunoblot with the indicated antibodies.

### Mass spectrometry

1D SDS-PAGE gel lanes were cut into 2-mm slices using an automatic gel slicer and subjected to in-gel reduction with dithiothreitol, alkylation with iodoacetamide and digestion with trypsin (Promega Corporation, USA, sequencing grade), essentially as described [54]. Nanoflow LC-MS/MS was performed on an 1100 series capillary LC system (Agilent Technologies, USA) coupled to an LTQ linear ion trap mass spectrometer (Thermo Fisher Scientific Inc., USA) operating in positive mode and equipped with a nanospray source. Peptide mixtures were trapped on a ReproSil C18 reversed phase column (Dr Maisch GmbH; column dimensions 1.5 cm×100 µm, packed in-house) at a flow rate of 8 µl/min. Peptide separation was performed on ReproSil C18 reversed phase column (Dr Maisch GmbH; column dimensions 15 cm×50 µm, packed in-house) using a linear gradient from 0 to 80% B (A = 0.1% formic acid; B = 80% (v/v) acetonitrile, 0.1% formic acid) in 70 min and at a constant flow rate of 200 nl/min using a splitter. The column eluent was directly sprayed into the ESI source of the mass spectrometer. Mass spectra were acquired in continuum mode; fragmentation of the peptides was performed in data-dependent mode. Peak lists were automatically created from raw data files using the Mascot Distiller software (version 2.1; MatrixScience). The Mascot search algorithm (version 2.2, MatrixScience) was used for searching against the SwissProt database (release SwissProt_54.8.fasta; taxonomy: mammalian). The peptide tolerance was typically set to 2 Da and the fragment ion tolerance was set to 0.8 Da. A maximum number of 2 missed cleavages by trypsin were allowed and carbamidomethylated cysteine and oxidized methionine were set as fixed and variable modifications, respectively. The Mascot score cut-off value for a positive protein hit was set to 60. Individual peptide MS/MS spectra with Mascot scores below 40 were checked manually and either interpreted as valid identifications or discarded. Typical contaminants, also present in immunopurifications using beads coated with pre-immune serum or antibodies directed against irrelevant proteins were omitted from the table. The proteins identified were further analyzed with the Ingenuity Pathway Analysis Software (Ingenuity Systems, Inc., USA) and clustered according to their involvement in different biological functions. The enriched categories obtained were represented according to their p-value.

### Chromatin Immunoprecipitation (ChIP)

5×10^8^ EC were fixed with 1% formaldehyde for 10 min at 37°C and subjected to quantitative ChIP analysis as previously described [55]. Briefly, 5 µg of the specific antibodies were pre-bound overnight, at 4°C, to protein G-Dynal magnetic beads (Invitrogen Corporation, USA), added to the diluted sonicated chromatin (4x 20 s, 50% output in Soniprep 150, Sanyo) and immunoprecipitated overnight, at 4°C. Antibodies used were as follows: VEGFR2 (Cell Signaling Tecnology, Inc., USA), Sp1 (Santa Cruz Biotechnology, USA), RNA Polymerase II (Covance, USA) and Rabbit/Mouse IgG (Santa Cruz Biotechonology, USA).

The magnetic bead-chromatin complexes were collected and washed in RIPA buffer (containing 50 mM HEPES pH 7.6, 1 mM EDTA, 0.7% Na deoxycholate, 1% NP-40, 500 mM LiCl). Chromatin-protein complexes were further washed in 1x TE buffer, eluted from beads in 1% SDS, 100 mM NaHCO3 and heated overnight at 65°C to reverse the formaldehyde cross-linking. DNA fragments were purified with a QIAquick Spin Kit (QIAGEN, Germany). The DNA amount of *VEGFR2* gene immunoprecipitated was quantified by RT-PCR using primers designed for the amplification of the *VEGFR2* proximal promoter (-300/-159 relative to the transcription start site). The primers used were as follows: 5′ CCGGCAAGCGATTAAATCTTGGAG 3′ (sense) and 5′ TTTCCCCACACAACTGGACTGC 3′ (antisense). Additionally were used primers for the amplification of an intergenic region in chromosome 10 as described [57]. The PCR reaction mixture used was as follows for a 25 µl total volume: 1x SybrGreen (Applied Biosystems, USA), 100 nM of each primer, and 2.5 µl of each ChIP DNA sample (input 1∶10). All reactions were performed and analysed as triplicates using a Fast 7500 Real time PCR (Applied Biosystems, USA). The results were normalized based on the {Delta}{Delta}Ct method as previously described [55], [56], [57]. Briefly, the threshold cycles (Ct) from total input samples were subtracted from the Ct of the IgG control and from the experimental IP (VEGFR2, Sp1 and RNA Pol II). The fold difference between the corrected value for the total input and corrected experimental IP value was calculated as 2{Delta}{Delta}Ct. The fold difference over background obtained for *VEGFR2* promoter was further normalized to the value obtained for the intergenic region.

### Quantitative RT-PCR

Total RNA and cDNA were prepared and quantitative RT-PCR (qRT-PCR) was performed as described [58]. *VEGFR2* primer sequences were as follows: 5′ ATTCCTCCCCCGCATCA 3′ (sense) and 5′ GCTCGTTGGCGCACTCTT 3′ (antisense).

*Sp1* primer sequences were as follows: 5′ TCGGATGAGCTACAGAGGCACAAA 3′ (sense) and 5′ AAAGTGCCCACACTCAGAGCTACA 3′ (antisense).

### Electroforetic mobility shift assays (EMSA) and Immunodepleting EMSA (IDEMSA)

The oligonucleotides including consensus recognition sequence for TFs YY1, NFkB and Sp1 are derived from Transcruz gel shift oligonucleotides (SantaCruz Biotechnology, USA). A DNA probe comprising the same region of the *VEGFR2* promoter (-300/-159) amplified in ChIP assays was also used. EMSAs were performed following standard methodology as described [10]. Probes were labeled with γ-ATP 32P (Perkin Elmer, USA) and incubated for 20 min at room temperature with 10 µg of EC nuclear extracts in a binding buffer (containing 10 mM HEPES, 4% Ficoll, 70 nM NaCl, 2 mM DTT, 100 µg/mL bovine serum albumin and 0.01% NP40). For competition assays, a 100-fold molar excess of unlabeled probe was incubated in the binding reaction. For supershift assays, 1 µg of the p65 or YY1 was added to the binding reaction for an additional 30 min at room temperature. DNA-protein complexes were resolved in 5% non-denaturating polyacrilamide gels. IDEMSAs were performed as described [59]. Briefly, 30 µg of EC nuclear extracts were depleted of VEGFR2 by immunoprecipitation with anti-human VEGFR2 antibody for 1 h followed by incubation with sepharose beads for an additional hour, at 4°C. After centrifugation, 10 µg of the VEGFR2 immunodepleted supernantant were used for each reaction of EMSA/Supershift as described above.

### Luciferase reporter assays

The proximal promoter of *VEGFR2* gene was amplified from genomic DNA by PCR and cloned into a pGL3-promoter firefly luciferase vector (Promega Corporation, USA). Briefly, the sequence from -300/+1 of the VEGFR2 proximal promoter was amplified using oligonucleotides with flanking restriction enzyme sites 5′ *Xho* I and 3′ *Bgl* II. The primers used were as follows: 5′ AAGCTCTCGAGGGTTAATTAAGACCGGCAAGCGATTAAATCTTGGAG 3′ (sense) and 5′ AGATCTTTAGATCTGTAGCAGGGTGGGAGCTGGTGCCGA 3′ (antisense). A deletion fragment of the region from −116/+1 bp of the *VEGFR2* promoter was obtained using the same procedure. The primer used was as follows: 5′ AGCTCTCGAGGGTTAATTAAGGTACCCGGGTGAGGGGCCGGGCT 3′ (sense). All constructs were confirmed by DNA sequencing. For luciferase reporter gene assays, NIH 3T3 GFP and NIH 3T3 VEGFR2-GFP expressing cells growing in 24-well plates were co-transfected with 400 ng of pGL3 control or pGL3 VEGFR2 (-300/+1) or pGL3 VEGFR2 (-116/+1) and 40 ng of the pCMV-βgal (Clontech Laboratories, Inc., USA) using the Fugene 6 transfection reagent (Roche Applied Science, USA). 24 h post-transfection, cells were harvested and luciferase activity was measured using Luciferase Assay Reagent (Promega Corporation, USA). β-galoctosidase activity was measured using Trophic Reagent (Applied Biosystems, USA). Results were normalized by dividing the luciferase activity values for β-galoctosidase activity values and represented as relative luciferase activity.

### Statistical Analysis

Data are represented as the mean ± s.e.m., and statistical analysis was performed with Student's *t* test. A *p* value <0.05 was considered statistically significant.

## Supporting Information

Figure S1 ***VEGFR2*** **and** ***Sp1*** **relative gene expression is decreased after VEGFR2 or Sp1 siRNA transfection.** EC were transfected with scrambled siRNA, VEGFR2 siRNA or Sp1 siRNA and 24 h later the *VEGFR2* or *Sp1* mRNA was quantified by qRT-PCR. Data are mean ± SD and represents the fold change in *VEGFR2* or *Sp1* gene expression relative to the internal calibrator (scrambled siRNA) in triplicates measurements and are representative of three independent experiments.
(TIF)

Figure S2 **Sp1 and VEGFR2 are present in the same DNA/protein complexes.** (A) Immunoprecipitation (IP) of 30 µg EC nuclear extract with anti-human VEGFR2 were analysed by immunoblot. The immunodepleted extract (ID VEGFR2) and Input were also included as control. (B) EMSA analysis of the VEGFR2 promoter with IgG-immunodepleted (ID Mouse IgG) (lane 3) or Sp1-immunodepleted (ID Sp1) extracts were conducted. As a positive control EC nuclear extracts (lane 2, 5) were also evaluated. Four complexes (C1–C4) are indicated with black arrows. Control lanes 1 and 4 contain only the radiolabeled probe.
(TIF)

Figure S3 **VEGFR2 binding to its own promoter is independent of EGFR activation.** ChIP assays of the VEGFR2 proximal promoter were performed using EC cultured in growing media and treated or not with 0.1 µM Iressa for 16 h. Ethanol was used as vehicle in the control cells. ChIP values are relative to control IgG background and normalized to an intergenic region. Data are mean ± s.e.m. of triplicates and represents three independent experiments.
(TIF)

Table S1 **VEGFR2‐interacting proteins as identified by MS analysis.** Proteins listed according to their Mascot score.
(XLS)
