# Supplementary figures and images for: VEGFR2 Translocates to the Nucleus to Regulate Its Own Transcription
Source: PLoS One. 2011 Sep 28;6(9):e25668. doi: 10.1371/journal.pone.0025668 (PMC3182252; doi:10.1371/journal.pone.0025668)

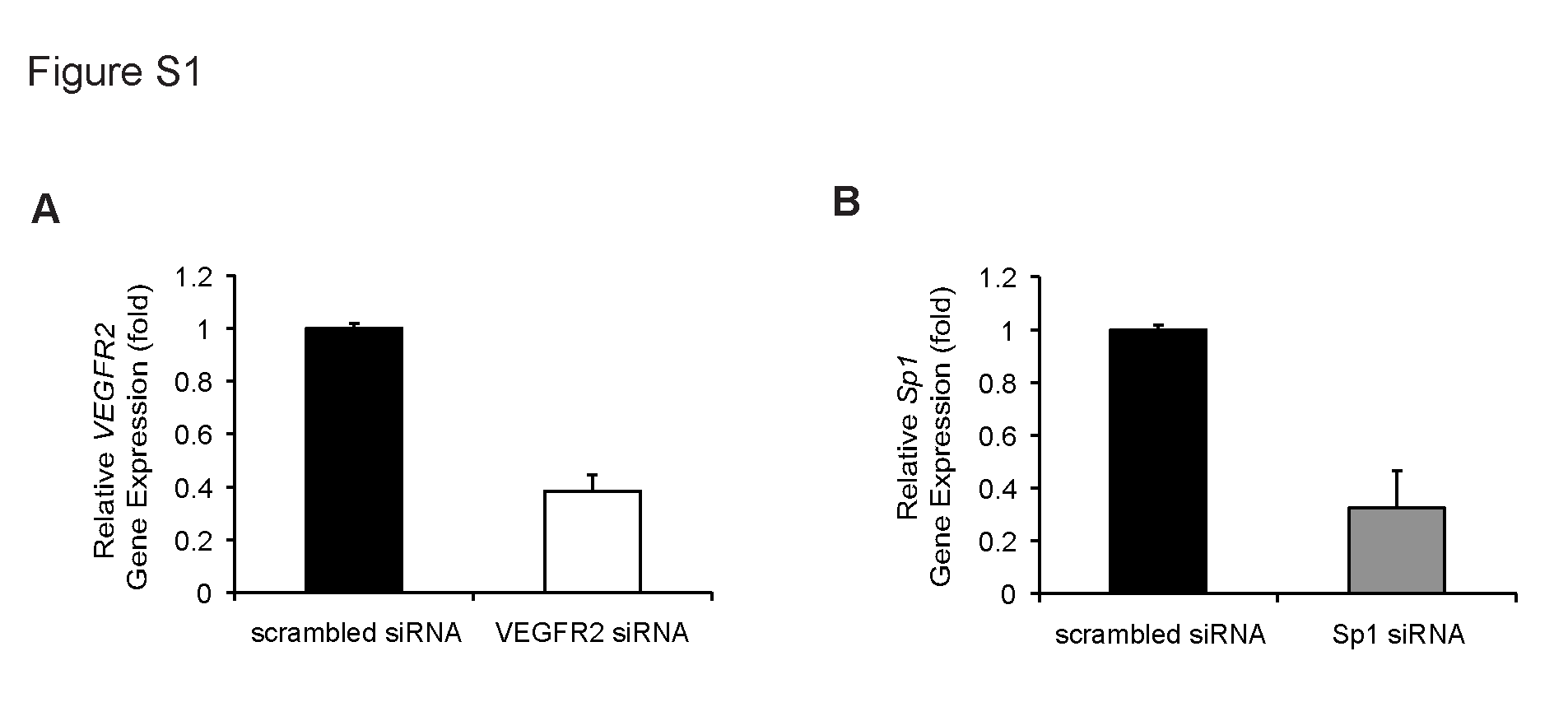

Supplement: Figure S1 — VEGFR2 and Sp1 relative gene expression is decreased after VEGFR2 or Sp1 siRNA transfection. EC were transfected with scrambled siRNA, VEGFR2 siRNA or Sp1 siRNA and 24 h later the VEGFR2 or Sp1 mRNA was quantified by qRT-PCR. Data are mean ± SD and represents the fold change in VEGFR2 or Sp1 gene expression relative to the internal calibrator (scrambled siRNA) in triplicates measurements and are representative of three independent experiments. (TIF) [file pone.0025668.s001.tif]

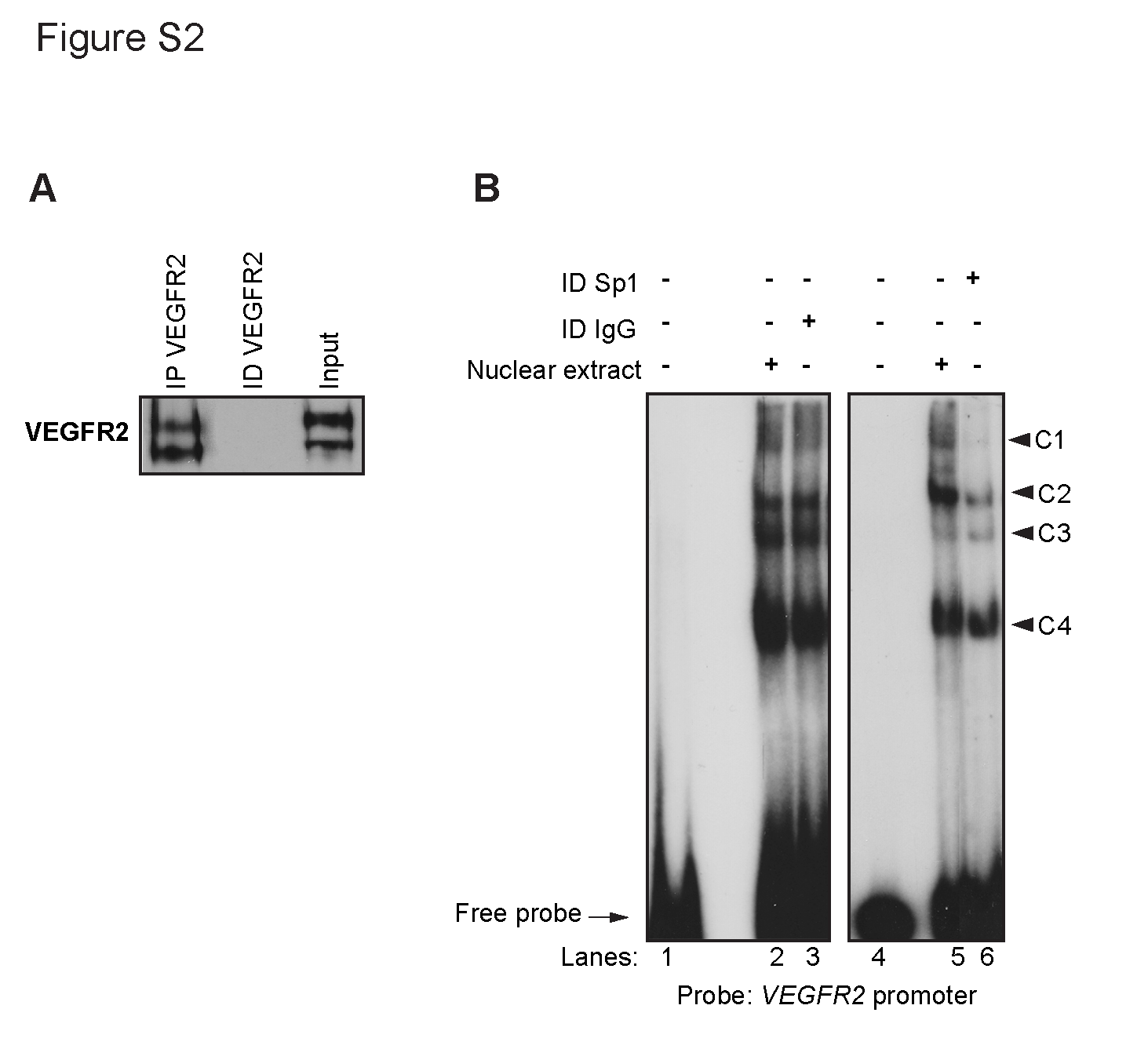

Supplement: Figure S2 — Sp1 and VEGFR2 are present in the same DNA/protein complexes. (A) Immunoprecipitation (IP) of 30 µg EC nuclear extract with anti-human VEGFR2 were analysed by immunoblot. The immunodepleted extract (ID VEGFR2) and Input were also included as control. (B) EMSA analysis of the VEGFR2 promoter with IgG-immunodepleted (ID Mouse IgG) (lane 3) or Sp1-immunodepleted (ID Sp1) extracts were conducted. As a positive control EC nuclear extracts (lane 2, 5) were also evaluated. Four complexes (C1–C4) are indicated with black arrows. Control lanes 1 and 4 contain only the radiolabeled probe. (TIF) [file pone.0025668.s002.tif]

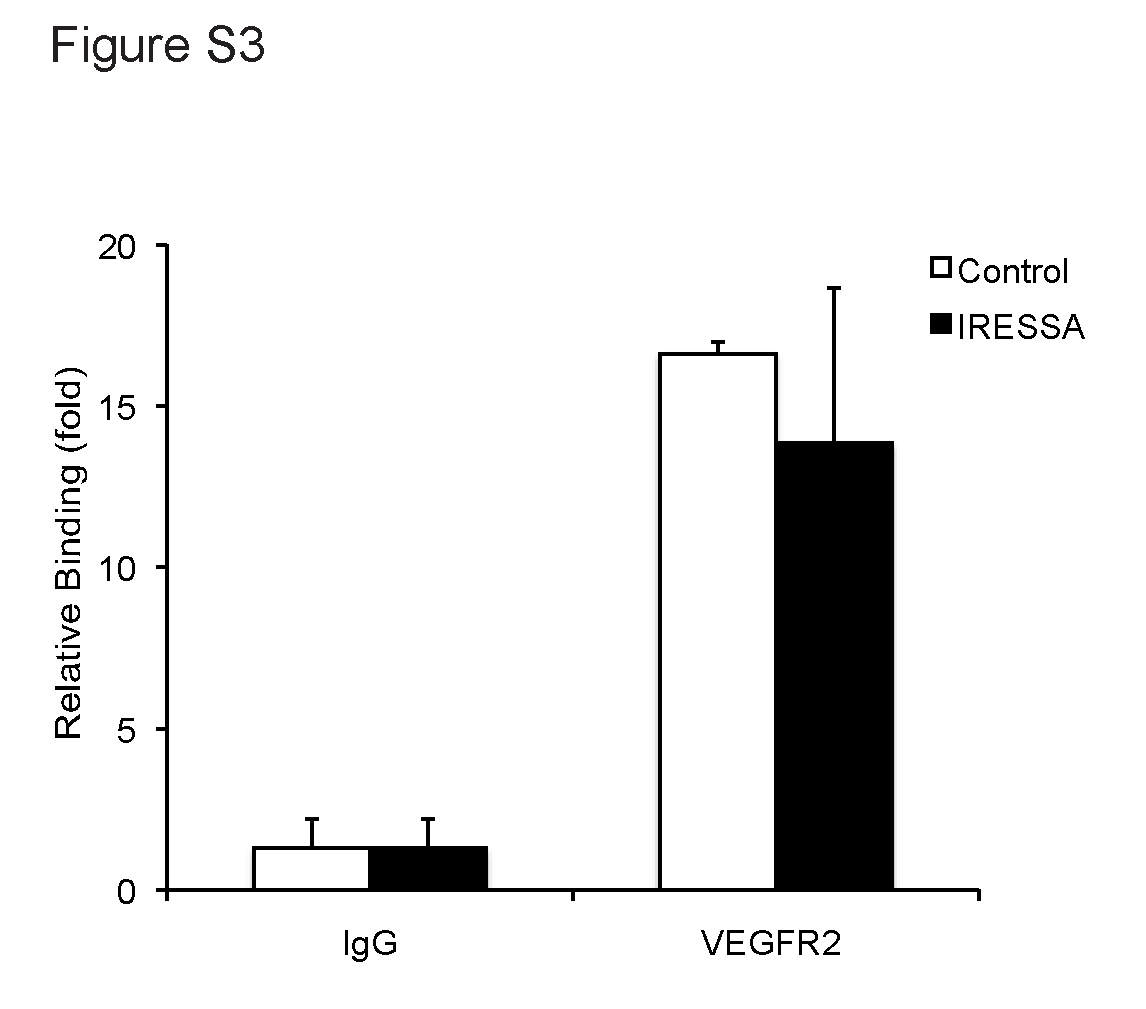

Supplement: Figure S3 — VEGFR2 binding to its own promoter is independent of EGFR activation. ChIP assays of the VEGFR2 proximal promoter were performed using EC cultured in growing media and treated or not with 0.1 µM Iressa for 16 h. Ethanol was used as vehicle in the control cells. ChIP values are relative to control IgG background and normalized to an intergenic region. Data are mean ± s.e.m. of triplicates and represents three independent experiments. (TIF) [file pone.0025668.s003.tif]
